# Lyme Disease, Virginia, USA, 2000–2011

**DOI:** 10.3201/eid2010.130782

**Published:** 2014-10

**Authors:** R. Jory Brinkerhoff, Will F. Gilliam, David Gaines

**Affiliations:** University of Richmond, Richmond, Virginia, USA (R.J. Brinkerhoff, W.F. Gilliam);; University of KwaZulu-Natal, Pietermaritzburg, South Africa (R.J. Brinkerhoff);; Virginia Department of Health, Richmond (D. Gaines)

**Keywords:** Acari, Borrelia burgdorferi, Ixodes scapularis, range expansion, zoonosis, ticks, Lyme disease, Virginia, vector-borne infections, bacteria

## Abstract

Lyme disease, caused by the bacterium *Borrelia burgdorferi* and transmitted in the eastern United States by the black-legged tick (*Ixodes scapularis*), is increasing in incidence and expanding geographically. Recent environmental modeling based on extensive field collections of host-seeking *I. scapularis* ticks predicted a coastal distribution of ticks in mid-Atlantic states and an elevational limit of 510 m. However, human Lyme disease cases are increasing most dramatically at higher elevations in Virginia, a state where Lyme disease is rapidly emerging. Our goal was to explore the apparent incongruity, during 2000–2011, between human Lyme disease data and predicted and observed *I. scapularis* distribution. We found significantly higher densities of infected ticks at our highest elevation site than at lower elevation sites. We also found that *I. scapularis* ticks in Virginia are more closely related to northern than to southern tick populations. Clinicians and epidemiologists should be vigilant in light of the changing spatial distributions of risk.

## Abstract 2

Geographic expansion of *Ixodes scapularis* ticks has increased human exposure to *Borrelia burgdorferi*.

Lyme disease (LD), caused by the bacterium *Borrelia burgdorferi* and transmitted in the eastern United States by the black-legged tick (*Ixodes scapularis*), is the most common vector-transmitted disease in North America (*1*). Maintained in an enzootic cycle comprising competent vertebrate reservoir host species, *B. burgdorferi* is transmitted to humans by the bite of an *I. scapularis* nymph or adult that acquired infection during a blood feeding as a nymph or larva (*2*). Although the principal reservoir host for this pathogen, the white-footed deer mouse, *Peromyscus leucopus*, is wildly distributed throughout North America, LD is generally confined to 2 geographic foci in the eastern United States: 1 in the upper Midwest and 1 in the Northeast (*2*–*5*). Densities of host-seeking *I. scapularis* nymphs correlate significantly with cases of human LD (*3*), but this species has been reported throughout much of eastern North America (*6*–*9*). Nationally, LD incidence increased during 1992–2002, but overall numbers of confirmed cases have since remained relatively stable (*1*,*10*).

In some locations, LD incidence recently has increased dramatically; in Virginia, the number of confirmed cases nearly tripled from 2006 to 2007 (http://www.vdh.virginia.gov/epidemiology/surveillance/surveillancedata/index.htm) to ≈12.4 cases per 100,000 residents, well above the 1998–2006 average of 2.2 per 100,000 (*1*). A 1990 report of LD cases in Virginia noted that the disease was rare in the early 1980s but apparently increased in incidence and geographic distribution through the late 1980s, leading the authors to conclude that the disease was expanding southward (*11*). Before 2006, most studies of *I. scapularis* ticks in Virginia focused on the eastern and southeastern parts of the state and found that densities of *I. scapularis* ticks declined, as did their rate of infection with *B. burgdorferi*, with distance from the coast (*12*,*13*). Several early surveys for *I. scapularis* ticks in Virginia’s neighboring states of North Carolina and Maryland also found them to be most abundant on the Coastal Plain but absent or less common in the Piedmont and Appalachian Mountains. During 1983–1987, Apperson et al. surveyed 1,629 hunter-killed deer from the Coastal Plain, Piedmont, and Appalachian Mountain regions of North Carolina and found *I. scapularis* ticks only on deer from the Coastal Plain (*14*). Amerasinghe et al. surveyed 1,281, and 922 hunter-killed deer in 1989 and 1991, respectively, at sites from the Coastal Plain to the Appalachian Mountains of Maryland and found *I. scapularis* ticks on 59%–70% of deer on the Coastal Plain, fewer on deer in the Piedmont Region, and on only 1%–5% of deer in the Appalachian Mountains (*15*,*16*).

Although *I. scapularis* ticks exist in the southeastern United States (*6*–*9*), they are most easily detected by drag sampling, a method used as a proxy for risk to tick exposure (*5*), in areas associated with highest LD incidence, i.e., the Northeast (New Jersey through Massachusetts) and upper Midwest (Wisconsin and Minnesota) (*3*,*5*,*17*). The difference in apparent abundance of *I. scapularis* ticks and risk for LD between the northern and southeastern United States has been the subject of much discussion and debate (*18*) and might be related, either through behavioral or physiologic mechanisms, to genetic differences between *I. scapularis* populations in these regions (*7*,*19*–*22*). Population genetic structure of *I. scapularis* ticks has shown that dynamic range shifts are likely to have occurred in recent evolutionary history (*19*–*22*) and that 2 distinct lineages within this species can be identified; a relatively genetically uniform “American clade” exists in the northern United States (although this lineage has also been detected in the South), and a genetically diverse “southern clade,” members of which have been found only in the South (*20*). Although other nomenclatures have been proposed for these 2 lineages (e.g., clades A and B for northern and southern lineages, respectively [*19*]), we follow the terminology established by Norris et al.: “American” describes the widely distributed yet less diverse clade and “southern” describes the geographically restricted yet more diverse mtDNA clade of *I. scapularis* ticks (*20*).

Range expansion of *I. scapularis* ticks over relatively short periods has been observed (*23*,*24*). Moreover, recent environmental modeling, based on extensive field collections of host-seeking *I. scapularis* ticks, suggests that this species suggests that the range of this species is expanding widely and its occurrence in a given area depends on the lack of abiotic drivers, vapor pressure deficit and elevation (*5*,*25*). In Virginia, studies found that *I. scapularis* ticks were concentrated in in northern sites; very few ticks were reported in other parts of the state (*5**,**17**,**25*). In contrast, human LD cases at inland, higher-elevation locations have increased in recent years in Virginia (http://www.vdh.virginia.gov/epidemiology/surveillance/surveillancedata/index.htm). The incongruity between human case and vector abundance datasets might be explained by recent (i.e., since 2007) spatial and/or numerical expansion of *I. scapularis* populations. We hypothesized that density of *B. burgdorferi*–infected ticks would be highest in counties associated with high incidence of human disease if epidemiologic data represent cases in tick-endemic areas. In contrast, low numbers of infected ticks in areas of high human disease might indicate either misdiagnosis or allochthonous exposure.

## Methods

### Data from Cases in Humans

We compiled LD cases reported to the Virginia Department of Health (http://www.vdh.virginia.gov/epidemiology/surveillance/surveillancedata/index.htm) directly by physicians or identified through follow-up of positive laboratory results by county public health department personnel during 2000–2011. All LD cases counted in Virginia State Reportable Disease Reports met clinical and laboratory criteria specified in the National Surveillance Case Definition (SCD) for LD. We assessed cases counted in Virginia during 2000–2007 using criteria in the 1996 SCD and cases counted during 2008–2011 using criteria in the 2008 SCD. The 1996 SCD enabled states to liberally interpret what constituted laboratory evidence of infection; an IgM-positive Western immunoblot (WB IgM) test result could be counted as laboratory evidence of infection even though a more specific 2-tier test that used an enzyme-linked immunoassay (EIA) and the WB IgM was recommended. The Virginia Department of Health used the less restrictive interpretation of laboratory evidence in its LD surveillance from 1996 through the end of 2007. However, given that single-tier positive results from either the EIA or the WB IgM are less specific than a positive 2-tier result from both the EIA and the WB IgM (*26*,*27*), laboratory evidence of infection in the 2008 SCD required, at a minimum, a positive 2-tier test result on blood collected during the acute phase of illness (i.e., within 30 days after illness onset). The more stringent laboratory criteria adopted in the 2008 SCD were designed to minimize the number of false cases counted by state surveillance programs.

We analyzed all data at the county level, which required us to reclassify cases reported in cities to the counties in which they are situated because cities and counties in Virginia are often separate administrative entities. We estimated LD incidence per county for each year during 2000–2011 by dividing the annual number of counted cases by the estimated population size in 2007 (*28*). To characterize annual change in incidence per county, we calculated the difference in cases between successive years and then averaged these values across years. We analyzed the spatial distribution of human LD cases at the state level by identifying the centroid, or geometric center, of county-level LD incidence for each year, starting in 2000 using ArcMAP 10.0 (ESRI, Redlands, CA, USA). We then used weighted linear regression to determine the effect of year on latitude and longitude of that year’s centroid position weighted by annual number of cases.

### Study Sites and Field Collections

**Figure 1:**
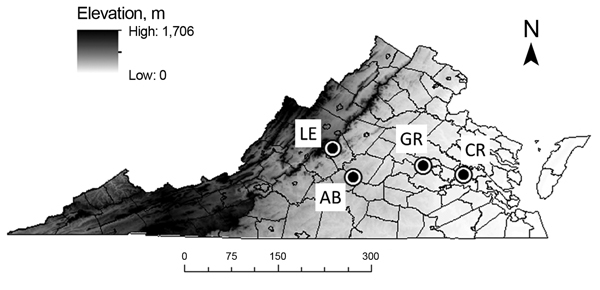
Locations of 4 field sites at which ticks were sampled, Virginia, May-July 2011. Circles indicate sampling areas. LE, Lesesne State Forest; AB, Appomattox-Buckingham State Forest; GR, University of Richmond–owned field site; CR, Crawfords State Forest. Darker shading represents higher elevation. Scale bar indicates kilometers.

In May and June 2011, we sampled ticks at 4 closed-canopy deciduous forest sites along an east-west elevational gradient: Crawfords State Forest (CR) (30 m), a University of Richmond–owned tract in Goochland County (GR) (80 m), Appomattox-Buckingham State Forest (AB) (170–200 m), and Lesesne State Forest (LE) (380–450 m) (Figure 1). We collected ticks at all sites by drag sampling (*29*) whereby a 1-m^2^ piece of corduroy was dragged along both sides of 5 haphazardly selected 100-m transects (1,000 m^2^ total), stopping every 20 m to remove ticks (*17*,*25*). We visited each site 4 times during May–July 2011 with at least 10 days separating visits. All ticks were speciated by light microscopy using dichotomous keys (*30*), and density of *I. scapularis* ticks was calculated as the average number of ticks collected per transect. Difference in density of *I. scapualris* nymphs among sites and visits was determined by analysis of variance of square root–transformed count data. We compared infection prevalence in ticks among sites by Gtest and by creating log-likelihood estimates of 95% CIs with a binomial probability function (*31*).

### Molecular and Phylogenetic Methods

To extract total DNA, individual ticks were dried and flash-frozen by using liquid nitrogen, crushed by using a sterilized pestle, and processed with Qiagen DNeasy Blood and Animal Tissue Kit (QIAGEN, Valencia, CA, USA) by using manufacturer’s protocols. We tested for *B. burgdorferi* DNA by PCR amplification of the outer surface protein C (*osp*C) gene and the intergenic spacer region of 16S–23S rRNA genes (*32*). Presence of amplified DNA was determined by gel electrophoresis, and samples that produced amplicons were purified with a QIAquick PCR Purification Kit (QIAGEN) and submitted for sequencing at the Nucleic Acids Research Facility at Virginia Commonwealth University (Richmond, VA, USA). We also performed PCR to amplify and subsequently sequence an ≈460-bp portion of the *I. scapularis* 16S rRNA gene using primers 16S +1 and 16S –1 (*20*). Bidirectional chromatograms from all sequence data were assembled and initially analyzed with Sequencher 4.10.1 (Gene Codes, Ann Arbor, MI, USA). *B. burgdorferi* sequences were blasted by using GenBank (http://blast.ncbi.nlm.nih.gov/Blast.cgi) to confirm species identification. Sequence data from *I. scapularis* 16S samples were aligned with reference sequences (*33*) by using ClustalW (http://www.clustal.org) implemented in MEGA 5.0 (http://www.megasoftware.net/), which was also used to select among models of evolution and to reconstruct phylogeny.

## Results

**Figure 2:**
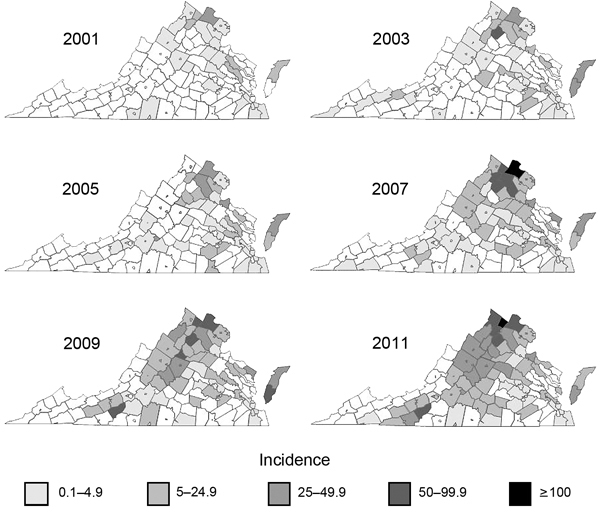
Progressive geographic spread of human Lyme disease across Virginia, 2001–2011. Data were reported by the Virginia Department of Health http://www.vdh.virginia.gov/epidemiology/surveillance/surveillancedata/index.htm. Cases per 100,000 population were calculated by county or city census estimate data published for the year preceding the year of the report. LE, Lesesne State Forest; AB, Appomattox-Buckingham State Forest; GR, University of Richmond–owned field site; CR, Crawfords State Forest.

During 1995–1998, the Virginia Department of Health counted 55–73 LD cases per year. The number increased to 122 cases in 1999, and cases continued to increase through the early 2000s. Although Virginia’s LD activity during 2000–2005 was focused primarily on northern Virginia and the Eastern Shore of Virginia (a peninsula extending south from Maryland on the eastern side of the Chesapeake Bay), small numbers of LD cases were recorded in counties across Virginia, including counties in the most southern and southwestern parts (Figure 2). During 2006–2007 the incidence of LD increased substantially in counties throughout the Appalachian Mountains (Figure 2). After the change in the SCD in 2008, many of the most southern and southwestern counties that had recorded LD cases before 2008 ceased to report cases, and the geographic progression of LD appeared as a compact front that progressed from county to county from northeast to southwest. LD cases were not observed again in any of the far southwestern counties until 2011, by which time LD was considered endemic to many of the counties immediately to their northwest (Figure 2).

**Figure 3:**
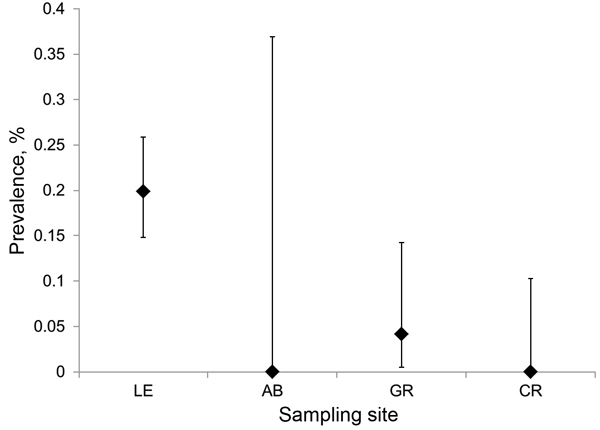
Variation in estimated prevalence of *Borrelia burgdorferi* infection in *Ixodes scapularis* nymphs at 4 field sites in Virginia. Sites are arranged west to east from left to right. LE, Lesesne State Forest; AB, Appomattox-Buckingham State Forest; GR, University of Richmond–owned field site; CR, Crawfords State Forest. Error bars represent 95% CIs.

We collected 2,549 ticks from the field: 2,192 *Amblyomma americanum* (1 larva, 1,917 nymphs, 274 adults), 306 *I. scapularis* (304 nymphs, 2 adults), 50 *Dermacentor variabilis* (all adults), and 1 *I. dentatus* (nymph). Sampling site was a major determinant of *I. scapularis* density (*F* = 71.07, p<0.0001, degree of freedom [df] = 3), as was sampling date (*F* = 6.85, p=0.024, df = 1). Post hoc comparisons indicated that tick density at the highest elevation site (9.55 nymphs/200 m^2^) was significantly greater than at any other site and that tick density at GR (1.66 nymphs/200 m^2^) was significantly higher than at site AB (0.25 nymphs/200 m^2^) (Table). We detected *B. burgdorferi* DNA in 48 *I. scapularis* nymphs, 45 of which produced unambiguous sequence reads for at least 1 locus (*osp*C or intergenic spacer region. Infection prevalence varied significantly among sites (likelihood ratio test, G = 16.3, p<0.0001, df = 3); the prevalence of infection was significantly higher at site LE (0.2) than at sites CR (0.00) and GR (0.04). Because of low sample size, site AB did not yield a reliable estimate of infection prevalence (Figure 3).

**Table:** Average density of host-seeking *Ixodes scapularis* nymphs, average prevalence of *Borrelia burgdorferi*–infected nymphs, and average annual LD incidence in 5 counties, Virginia

County	Nymphs				Humans	
	Average density (SEM)†	Average prevalence	Average incidence of *B. burgdorferi* infection, 2000–2007		Average LD Incidence, 2008–2011‡	Average yearly change in LD cases, 2008–2011
Nelson	9.55 (1.30)	0.20	0		32.3	+9.7
Appomattox-Buckingham	0.25 (0.11)	0	0.87		4.59	+2.6
Goochland	1.66 (0.33)	0.06	0		0	0
New Kent	1.1 (0.21)	0	0.83		0	0

*LD, Lyme disease. †Nymphs per 200 m^2^. ‡Per 100,000 persons.

**Figure 4:**
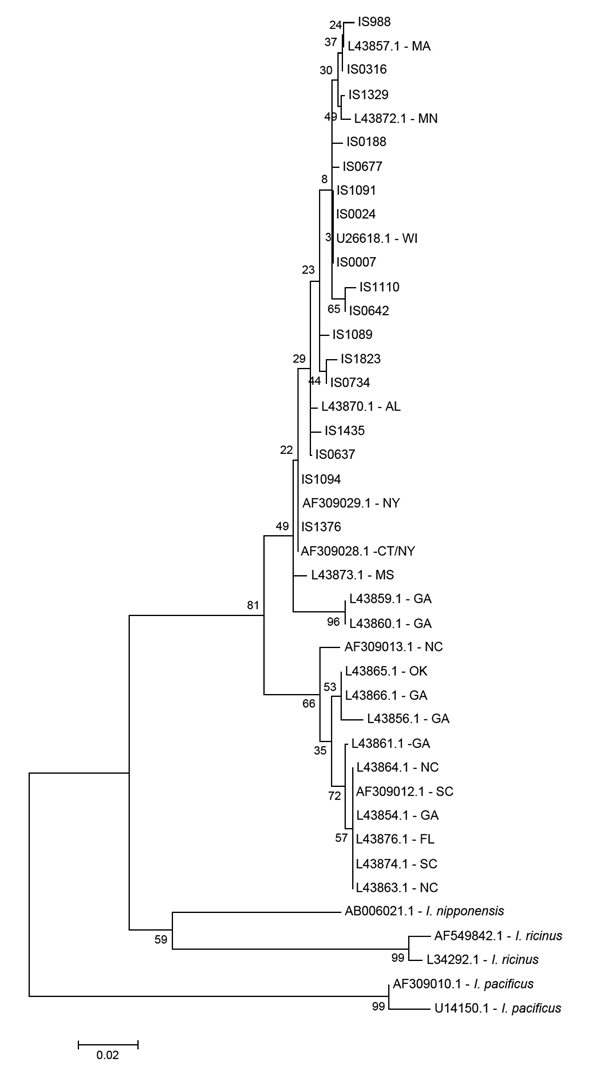
Maximum-likelihood phylogenetic reconstruction of *Ixodes scapularis* lineages based on 16S rRNA gene sequences using Tamura 3-parameter model (*35*). All samples beginning with IS were collected during this study; reference sequence GenBank accession numbers are indicated, as were sampling locations (2-letter state abbreviation). The clade containing samples collected in GA, FL, NC, OK, and SC is known as the Southern clade (sensu Norris et al. [*20*]); the clade containing all samples from this study, indicated by the prefix IS, represents the American clade (more complete explanation of these terms is provided in the text). Bootstrap values at nodes are based on 500 replicates. Scale bar indicates nucleotide substitutions per site.

**Figure 5:**
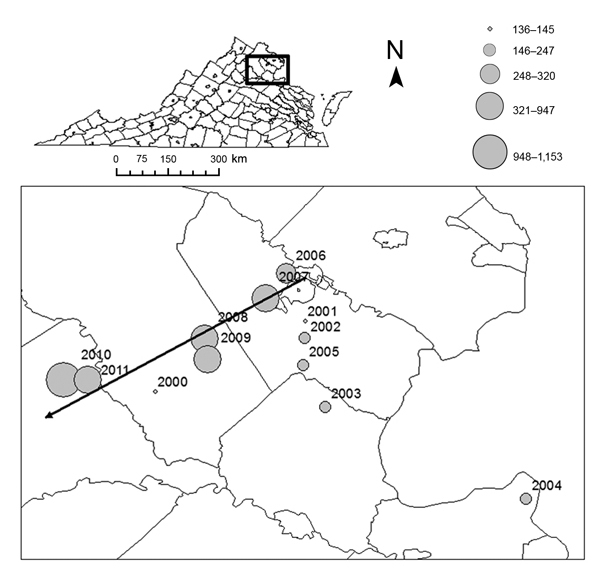
Centroids of annual incidence, by county, Virginia, 2000–2011. The size of each circle represents the annual number of cases reported by the Virginia Department of Health and is proportional to annual incidence (cases/100,000 population). Black arrow represents the mean linear direction of annual movement among centroids during 2006–2011 (these years indicate the recent dramatic increase in Lyme disease incidence in Virginia).

Analysis of *I. scapularis* 16S sequences yielded 17 haplotypes (GenBank accession nos. KF146631–47) from 85 individual nymphs (14 haplotypes from 44 ticks at LE, 1 from 2 ticks at AB, 6 from 21 ticks at GR, and 4 from 18 ticks at CR). Maximum-likelihood phylogenetic reconstruction using Tamura 3-parameter model (*34*) indicated that all haplotypes detected fall within the American clade; none of the ticks we sampled were phylogenetically identified as southern clade *I. scapularis* (Figure 4). In addition to an overall increase in human LD cases (from 136 in 2000 to an average of >1,000 in 2010 and 2011), we observed a significant spatial shift of the geometric center of LD incidence in Virginia. The longitude value associated with the centroid of each year’s LD incidence depended significantly on year from 2000 to 2011 (*F* = 12.48, p = 0.005, r^2^ = 0.56) (Figure 5). Latitude values did not change significantly over time (*F* = 0.14, p = 0.71, r^2^ = 0.01). We also calculated the average LD incidence per county for 2000–2006 (before the dramatic spike in cases in Virginia) and for 2007–2011 to identify counties in which the largest increases in cases occurred (Table).

## Discussion

Our results indicate that 1) human LD incidence in Virginia has increased since 2000 and that the spatial distribution of cases has changed significantly, 2) abundance of *I. scapularis* nymphs and prevalence of *B. burgdorferi* infection are consistent with recent changes in human disease data, and 3) *I. scapularis* populations detected in central and western Virginia are dominated by American-clade haplotypes. Taken together, these results suggest recent spatial and/or demographic expansion of *I. scapularis* ticks in Virginia, resulting in increased human exposure to *B. burgdorferi*; the most notable increases in ticks and disease risk are at higher elevations in the western part of Virginia. More generally, our results indicate a dynamic pattern of LD risk. The spatial trends we identified through acarologic sampling are consistent with observed changes in disease incidence and are of paramount public health importance; the observed changes LD epidemiology in Virginia most likely reflect a spatial increase in disease endemicity (Table). We propose that the increase in LD in Virginia is caused by either increasing abundance of *I. scapularis* ticks, increasing prevalence of *B. burgdorferi* infection in the vector, or both. Our data suggest that this vector species may be more abundant than it was before 2007; during widespread collections during 2004–2007, *I. scapularis* ticks existed throughout most of Virginia, and no infected *I. scapularis* ticks were detected in central or western Virginia (*17*,*25*). Similar range expansion of *I. scapularis* ticks has been described in Wisconsin and Michigan (*23*,*24*).

The extent to which the spatial distribution of LD cases in Virginia will continue to change is unclear. Environmental variables previously identified as important drivers of *I. scapularis* abundance may not have uniform effects throughout the range of this species. For example, on the basis of extensive sampling in the eastern United States over several years, Diuk-Wasser et al. estimated an elevational threshold of 510 m for this species (*25*), and Rosen et al. detected more *I. scapularis* on deer at low elevation than high elevation sites in Tennessee (*35*). However, our sampling showed the highest density of host-seeking *I. scapularis* nymphs at elevations approaching this threshold, and we have subsequently collected host-seeking nymphs at >1,000 m in Nelson County in west-central Virginia (R.J. Brinkerhoff, unpub. data). In 2007, a growing focus of LD incidence was observed in southwestern Virginia in Pulaski, Floyd, and Montgomery Counties. These counties have continued to have that region’s highest incidence of LD through 2011 (http://www.vdh.virginia.gov/epidemiology/surveillance/surveillancedata/index.htm) and mostly occupy high mountain valleys with average elevations of 584–762 m. An elevational threshold that limits tick populations at northern latitudes, where high elevation sites experience extreme cold during winter months, would not be expected where equivalent elevations are associated with more moderate climatic conditions.

Our analysis of LD data from humans indicates that the largest increases in LD incidence since 2007 has occurred in higher-elevation counties in western Virginia; the correspondence between these data and acarologic sampling suggests that the cases reported in these locations most likely are locally acquired and indicate recent spatial and/or numerical expansion of human disease. Our results are notably inconsistent with the findings of surveys of *I. scapularis* ticks on hunter-killed deer in North Carolina and Maryland during 1987–1992, which indicated that *I. scapularis* ticks were most abundant on the Coastal Plain and absent or uncommon in the Appalachian Mountains (*14*–*16*). When human LD surveillance began in Virginia in 1989, the highest incidence was on the state’s Eastern Shore (http://www.vdh.virginia.gov/epidemiology/surveillance/surveillancedata/index.htm). This finding was consistent with early surveys of ticks indicating that *I. scapularis* was the most common species in the Coastal Plain and much less common at higher elevations to the west (*14*). A logical conclusion at that time was that LD would continue to spread southward along the state’s Coastal Plain. However, during 2000–2011, LD became more prevalent in Virginia’s upper Piedmont and Appalachian Mountain zones than in the lower Piedmont and Coastal Plain. The results of older surveys of ticks and recent environmental models are not consistent with the current geographic incidence of LD or our field data. This discrepancy suggests a southwestward spatial expansion of northern tick populations into the upper Piedmont and mountain regions of Virginia or demographic expansion of persons into areas of previously low tick density in western localities. We do not have acarologic data from each county in which LD incidence has increased, nor do we have long-term systematic sampling data, and thus we cannot directly attribute local changes in LD to changes in tick densities.

Analysis of single-nucleotide polymorphisms in the *I. scapularis* genome reinforces the hypothesis that these ticks recolonized northern North America after the most recent glaciation event and that northern populations are genetically less diverse than southern populations (*21*). Moreover, analyses of single-nucleotide polymorphism data are consistent with south-to-north postglaciation gene flow, whereby northern American-clade populations are a subset of the genetic variation found in southern-clade populations (*21*) resulting from founder effects when ticks recolonized northern latitudes (*22*). Tick populations within both LD-endemic foci show signs of genetic isolation from one another and from southern populations (*22*), and evidence exists for similar lack of gene flow among populations within regions (*19*). Identification of American-clade *I. scapularis* ticks in the southeastern United States (*19*,*33*) might reflect remnant American-clade lineages in the South or might indicate southward dispersal of American-clade ticks. Qiu et al. noted that coastal sites in southern states were associated with strictly American-clade populations, whereas a mix of American- and southern-clade ticks was detected at inland sites (*19*). With respect to our study, we point to the recent lack of detection of *I. scapularis* ticks at high-elevation sites in western or central Virginia (*17*,*25*) and the presence of exclusively American-clade *I. scapularis* ticks in the current study as possible evidence consistent with the population expansion of American-clade ticks from northern population foci. However, we cannot exclude the possibility that the distribution of endemic American-clade ticks simply has expanded in Virginia.

Although American- and southern-clade *I. scapularis* ticks are now considered 1 species, apparent differences exist in host-seeking behavior, biting behavior, and duration of attachment to different host types (*9*,*36*,*37*). Genetic differences between the major *I. scapularis* lineages have been well documented (*7*,*19*–*22*), and if American-clade ticks are more likely to feed on humans, the emergence of LD in Virginia would be consistent with increased relative abundance of this variant. In the South, immature *I. scapularis* ticks feed predominantly on low-competence or noncompetent lizard species and are relatively uncommon on rodents (*8*,*36*–*38*). Southern-clade nymphs may have questing behavior that makes them unlikely to be collected on cloth drags or to bite humans (*9*); thus, nymphal ticks are difficult to collect, even in places where adult ticks are common. LD risk should be very low in areas where *I. scapularis* nymphs are unlikely to bite humans, and immature ticks are more likely to feed on reptiles than on competent vertebrate reservoirs. However, data from a single mitochondrial gene, albeit one that has been widely characterized for this species, do not necessarily reflect patterns of differentiation found in nuclear markers (*21*) and probably are not useful for delineating among behavioral phenotypes. Moreover, we sampled in daytime hours during the presumed peak period of nymphal activity (late spring, early summer) and thus would not have detected ticks exhibiting different host-seeking behaviors. It is possible that multilocus genomic analysis or year-round sampling would yield different insights from those reached in this study.

The latitudinal gradient in LD risk in the eastern United States is not easily explained and probably is driven by demographic and environmental factors (*5*,*26*,*39*). However, our data suggest that the boundary between regions to which *I. scapularis* ticks are and are not endemic is moving and that *B. burgdorferi*–infected ticks might be expanding in or into areas from which they historically have been absent. As a result, clinicians and epidemiologists need to be vigilant in the face of changing spatial distributions of risk, especially in transition zones where patterns of disease are rapidly changing (*40*).
